# Predictors of alcohol and other drug use among pregnant women in a peri-urban South African setting

**DOI:** 10.1186/s13033-016-0070-x

**Published:** 2016-05-04

**Authors:** Michael Nnachebe Onah, Sally Field, Thandi van Heyningen, Simone Honikman

**Affiliations:** Perinatal Mental Health Project, Alan J. Flisher Centre for Public Mental Health, Department of Psychiatry and Mental Health, University of Cape Town, Cape Town, South Africa

**Keywords:** Alcohol abuse and drug use, AOD, Poverty, Pregnancy, Maternal mental health, Depression, Anxiety

## Abstract

**Background:**

Alcohol and other drugs (AOD) use among pregnant women have been associated with adverse health outcomes for mother and child, during and after pregnancy. Factors associated with AOD use among women include age, poverty, unemployment, and interpersonal conflict. Few studies have looked at demographic, economic, and psychosocial factors as predictors of AOD use among pregnant women in low-income, peri-urban settings. The study aimed to determine the association between these risk factors and alcohol and drug use among pregnant women in Hanover Park, Cape Town.

**Methods:**

The study was undertaken at a Midwife Obstetric Unit providing primary-level maternity services in a resource-scarce area of South Africa. 376 adult women attending the unit were recruited and a multi-tool questionnaire administered. Demographic, socioeconomic and life events data were collected. The Expanded Mini-International Neuropsychiatric Interview Version 5.0.0 was used to assess alcohol abuse and other drugs use, depression, anxiety, and suicidal ideation. Descriptive and bivariate analyses were conducted to examine the associations between predictor variables. Non-parametric tests, Wilcoxon sum of rank test, Fisher Exact and two sample *T* test and multicollinearity tests were performed. Logistic regression was conducted to identify associations between the outcome of interest and key predictors. A probability value of p ≤ 0.05 was selected.

**Results:**

Of the total number of pregnant women sampled, 18 % reported current AOD use. Of these, 18 % were currently experiencing a major depressive episode, 19 % had a current anxiety diagnosis, and 22 % expressed suicidal ideation. Depression, anxiety, suicidality, food insecurity, interpersonal violence, relationship dynamics, and past mental health problems were predictors of AOD use.

**Conclusions:**

This study has confirmed the vulnerability of pregnant women in low-income, peri-urban settings to alcohol abuse and other drugs use. Further, the association between diagnosed depression and anxiety, suicidality, and AOD use among these women may reflect how complex environmental factors support the coexistence of multiple mental health problems. These problems place mothers and their infants at high risk for poor health and development outcomes. The results have implications for planning appropriate interventions.

## Background

Alcohol and other drugs (AOD) use among pregnant women has been identified as associated with adverse health outcomes for mother and child both during and after pregnancy [1–3]. AODs has also been identified as a means for ‘coping with everyday life’ for pregnant women in adverse environments where poverty and other societal harms exist [2, 4]. Exposure to alcohol during pregnancy has been identified as one of the main avertable causes of birth defects and developmental impairment in offspring [5]. Frequent AOD use has also been identified as associated with low weight gain during pregnancy [6], diminished fetal growth [7], and premature deliveries [5]. Studies have found that South Africa has one of the highest prevalence rates for Fetal Alcohol Spectrum Disorders (FASD) in the world [8, 9]. A study in South Africa, using urine analysis, found the prevalence of drug and alcohol use among pregnant women attending antenatal clinics in Cape Town to be 8.8 and 19.6 % respectively [9]. The 2010/2011 snap-shot survey report on substance abuse in the nine provinces in South Africa, indicated that the use of drugs was twice the global average and that South Africa was amongst the top ten nations in high alcohol consumption [10].

AOD use have been found to be associated with age [11], poverty [12, 13], unemployment [14, 15], interpersonal conflict [16, 17], multiple depressive episodes [18], anxiety [19], suicidality [20] among pregnant women. Low income pregnant women have higher vulnerability to AOD use in both developed and developing country settings [11, 12, 21]. Studies in high income countries have shown that there is a strong correlation between low socioeconomic status, minority race, and AOD use among pregnant women [22], indicating that older black women with average incomes consume more alcohol than their white counterparts in the USA. However, younger white women consume more than their female black counterparts of the same age. There is evidence of a strong association between poverty and drug use among pregnant women in the USA [23] disaggregated by race.

In South Africa, studies have examined alcohol use among pregnant women [24, 25] but less data is available on drug use [9]. Few studies have looked at the association between hazardous behaviour, demographic, and socioeconomic factors among pregnant women [26]. Studies have predominantly focused on the impact on FASD and health outcomes of children [1, 3, 27, 28] with few studies having looked at the relationship between AOD use and life events among pregnant women [29]. Most studies have however disaggregated data into racial categories [14, 22, 27]. A study in South Africa analysed the predictors of alcohol and drug use among pregnant women using screening tools; the Alcohol Use Disorders Identification Test (AUDIT) and the Drug Use Disorders Identification Test (DUDIT) [30]. However, the study did not use a diagnostic tool to determine alcohol and drug use and also did not perform multiple regression analysis.

The aim of this study is to determine the association between demographic, economic and psychosocial life events with alcohol and drug use among pregnant women in Hanover Park, Cape Town using The Expanded Mini-International Neuropsychiatric Interview (MINI Plus) Version 5.0.0. as the diagnostic interview for mental health disorders.

## Methods

### Ethics

The Western Cape provincial Department of Health and the University of Cape Town Human Research and Ethics Committee provided ethical approval for the study (HREC REF: 131/2009). Written informed consent was obtained from all respondents after the study was verbally explained. The consent forms were available in English and the local languages.

### Setting

This study was a cross-sectional study and was conducted at the Hanover Park Midwife Obstetric Unit (MOU). This facility provides maternity services in the residential and semi-industrial urban area of Hanover Park in Cape Town, South Africa. Hanover Park has a population density of 35,000 in an area of approximately two square miles, which is the highest population density in Cape Town [31]. It is considered one of the most violent communities in Cape Town, with high levels of poverty, gang-related violence, alcohol and drug abuse, and physical and sexual abuse [32, 33]. Residences comprise of overcrowded, public apartment units erected in the Apartheid era, with poor infrastructure such as toilets and plumbing. There are also small free-standing houses and an increasing number of informal dwellings. High levels of adult illiteracy are prevalent within the community as less than 20 % of the adult population completed high school. This has resulted in low rates of regular employment and income [33].

At the time of this study, there was no mental health service in Hanover Park for pregnant women in particular (personal communication with the Hanover Park Community Health Centre Manager, 01 May, 2010). Even though these patients were theoretically able to access general mental health outpatient and social work services at the Hanover Park Community Health Centre (CHC), referral to these overburdened services almost never took place. Psychiatric emergencies were referred to the CHC’s casualty unit or to tertiary level hospitals.

### Sample

Participants were recruited by systematic sampling of every third woman arriving at the Hanover Park MOU for her first antenatal visit. This strategy was used after considering the average number of women that attended the MOU for antenatal care and the duration of time spent on their screening. This information was then used to estimate the average number of women screened on a daily basis and for calculating the sample number. This technique ensured that participant recruitment and data could be feasibly gathered with a cross-section of women that presented themselves at the MOU all through the day. Women recruited did not undergo any initial medical evaluation. Consent was sought from women that were 18 years and above, pregnant, and willing to participate in the study. A total of 376 women were interviewed.

### Measures

Demographic and socioeconomic information was collected using a questionnaire that assessed the age, language, education, marital status, socioeconomic status (SES), obstetric information, whether the pregnancy was planned, wanted, as well as past psychiatric history. Information on participants’ monthly income was collected and converted to United States Dollars.^1^

To measure the socio-economic status of the women recruited, an asset index was constructed using information on household ownership of electronic equipment (e.g., microwave, washing machine, television and fridge), transport (cars, motorbike, bicycle), sources of energy (electricity), and bank accounts (including credit card). The use of asset indexes in the measure of household socioeconomic status was chosen over the use of total household income because income data collection has numerous methodological imperfections in low-income settings. These include the difficulty with collecting such data, problems of recall bias, and its lack of sensitivity to non-cash income [34–37]. While constructing the index using a principal component analysis, the first component factor.^2^ was used to represent the asset index. The study sample was then stratified into 4 quintiles (i.e., least poor, poor, very poor and poorest). Food insecurity was examined using the revised six-item Household Food Security Survey Module (HFSSM) [38]. This tool collected information from a period six months prior to the survey. The scale uses a 6-question checklist to ascertain food insecurity and food insufficiency. A score of 0–1 is considered food secure, 2–4 food insecure, while 5–6 is labelled as food insufficient (a more severe form of food insecurity) [38, 39].

The perceived social support from family, friends and a significant other was assessed using the Multidimensional Scale of Perceived Social Support (MSPSS). The MSPSS displays good psychological measurement precision in different study samples, displays a good internal reliability, and a strong factorial validity [40]. This measure has been used previously in South African populations [15, 41]. The tool consists of scores which range between 12 and 84 for the whole instrument and between 4 and 28 for three sub-scales. While there are no exact points for measurement, higher scores on the scale indicate an increase in perception of support.

To assess for the presence of risk factors for psychological distress during pregnancy, the Risk Factor Assessment (RFA) was used. The RFA was developed by the Perinatal Mental Health Project [42], based on their local clinical practice and the common risk factors for perinatal psychological distress and depression identified in the literature [43, 44]. It contains of a list of 11 items, each measuring the presence or absence of one risk factor, with a yes or no response option [45].

The revised conflict tactic scales (CTS2) was used to assess intimate partner violence (IPV) amongst pregnant women in peri-urban settlements in South Africa. The CTS2 is a condensed form of the original conflict tactics scale (CTS) and are used in low-income countries which are resource-constrained to screen for inter-personal violence amongst women [46]. This tool has a good reliability and has been used in cross-cultural studies in South Africa [47, 48].

This study used a widely accepted diagnostic tool, the Expanded Mini-International Neuropsychiatric Interview (MINI Plus) Version 5.0.0 to assess information on common mental disorders (major depressive episode (MDE), and anxiety disorders), suicidality, alcohol abuse and drug use [49]. The MINI Plus is relatively quick and easy to administer, and contains components for the major axis-I psychiatric conditions in DSM-IV TR. This diagnostic tool has been validated for application in South Africa [50] and is available in English and the local languages spoken by the women attending the MOU [51, 52]. Alcohol and drug use data were each collected separately but due to low statistical power emanating from low reporting of usage, the data were combined to form an alcohol and other substance (AOD) use measure. AODs terminology has been used extensively in literature to connote alcohol and other drugs use [53, 54].

## Procedures

### Training

A clinical psychologist, with research experience, trained and supervised a research assistant and mental health officer that were responsible for recruitment and screening, and the diagnostic interviewing respectively. The mental health officer holds a 4-year undergraduate degree in psychology and is registered with the Health Professions Council of South Africa as a counsellor. The mental health officer was trained to administer the diagnostic interview and to provide counselling to women who indicated mental distress in the interview process or who qualified with any MINI-defined diagnosis. A referral protocol between the research staff, MOU and CHC was established for women that presented more severe mental health problems and required higher-level psychosocial interventions. A pilot study was conducted to assess how feasible and acceptable the research protocol was for both participants and clinic staff.

### Data collection

Data were collected at the Hanover Park MOU between 22 November 2011 and 28 August 2012. Retrospective HIV status data was collected from the women’s hospital records. HIV testing was a routine part of the antenatal appointment which occurred after the study interviews were complete.

### Data analysis

Univariate, bivariate, and multivariable analyses was performed using Stata v13.1. The univariate analysis produced descriptive statistics which described the data using sample statistics. Significant associations between alcohol and other drug use (AODs), demographic factors, socioeconomic factors, and psychosocial risk factors, Major Depressive Episode (MDE), anxiety disorder diagnoses and suicidal ideation were examined using non-parametric, Wilcoxon sum of rank, Fisher exact and two sample t-tests. A univariate analysis was conducted for the assets owned and other factors that were included in the asset index. To ensure that results were not skewed in the principal component analysis, all assets that were identified (or not) by the majority of the respondents were not included in the analysis. The 95 % confidence interval was selected as the level of significance.

Bivariate analyses were conducted to examine the associations between predictor variables. Variables which were significant at a probability value (p value) equal to or less than 0.05 were selected and included. Internal consistency and scale reliability within assessment tools were assessed using the Cronbach’s α Statistics [55]. Logistic regression was conducted to identify associations between the outcome of interest and key predictors, with results presented as odds ratio (OR). The dependent variable was AODs use and the independent variables included age, education level, food insecurity and insufficiency, asset index, employment status, life events, interpersonal violence, planned pregnancy and relationship types, MDE and anxiety diagnoses, suicidal ideation. Mini logistic models were used to test the associations between the dependent and independent variables and all the preliminary variables were included in an OLS (ordinary least squares) model to test for multicollinearity. Likelihood ratio tests were used to assess nested models and to test for significance. All items with a statistically significant bivariate association at p  ≤  0.05 were included in the final multivariable model.

## Results

### Description of the pregnant women sampled

On average, 39 % of women were in their mid-twenties (25–29 years old), and over half of the women sampled were in weeks 12–28 of pregnancy (Table 1). While the majority of women were in a relationship (94 %), only 39 % were married and over half (51 %) in a stable relationship. Sixty percent of those that were in a relationship were living with their partner, 28 % were not, and 12 % partly cohabited with their partners. The average maximum level of education attained by the study participants were Grade 10 (US 10th grade), and over half of these women (58 %) were unemployed. Of those that earned an income, only 5 % earned over R5000 (US$500) a month. The asset index indicated that 29 % of women belonged to the highest socioeconomic status (least poor), while 23 % belonged to the lowest (poorest). Forty-two percent of the sampled women were food insecure while 12 % were food insufficient. In total, 18 % of women sampled reported current alcohol abuse and/or drug use. Of these, 76 % abused alcohol while 24 % used drugs. Seventy-three cases of alcohol or drug use were reported among 65 women indicating that 8 women reported both alcohol and drug use. Although retrospective data indicated an HIV prevalence of 11 % among sampled participants, HIV status was not assessed at the time of data collection.

**Table 1:** Demographic and socio-economic characteristics of participants

Variable	N (376)	Percentage
Alcohol and other substances use		
Positive	65	18
Negative	311	82
Demographic factors		
Age of woman		
18–24 years	146	39
25–29 years	114	30
Above 29 years	116	31
Gestation in weeks		
First trimester	96	32
Second trimester	175	58
Third trimester	29	10
Relationship status		
In a relationship	354	94
Single/not in a relationship	22	6
Type of relationship		
Married	144	39
Stable relationship	192	51
Casual relationship	16	4
No relationship	22	6
Cohabiting with partner		
No	97	28
Yes	209	60
Sometimes	44	12
Education level		
Grade 10 or below	151	40
Higher than grade 10	225	60
Socio economic factors		
Employment status		
Working currently	159	42
Not working currently	217	58
Asset index		
Least poor	107	29
Poor	91	24
Very poor	92	24
Poorest	85	23
Individual income (monthly)		
R0 ($0)	97	26
R1–R1000 ($1–100)	99	26
R1001–R2000 ($101–200)	64	17
R2001–R5000 ($201–500)	97	26
>R5000 ($500)	19	5
Food security		
Food insecure	158	42
Food insufficient (extreme food insecurity)	50	13

Results from the bivariate analyses (Table 2) indicated that out of the total number of women who abused alcohol and/or used drugs, 18 % were currently experiencing a MDE, 19 % had a current anxiety diagnosis, and 22 % expressed suicidal ideation. Suicidality was assessed as high, medium, and low risks. Out of the 68 women (18 % of the total study sample) that reported any suicidal ideation, 45 measured high suicidal risk, 9 medium, and 14 low suicidal risks.

**Table 2:** Bivariate associations between AOD use, MDE, any anxiety diagnosis, and suicidal ideation

	AOD use (n = 65) (%)	No AOD use (n = 311) (%)	p value
Major depressive episode (MDE) (n = 81)	37	18	0.001
Any anxiety disorder (n = 86)	37	19	0.003
Suicidal ideation (n = 68)	32	15	0.001
High suicidal ideation (n = 45)	22	11	0.025
Medium suicidal ideation (n = 9)	8	2	0.023
Low suicidal ideation (n = 14)	11	4	0.031

Although 42 % of women sampled were employed, 23 % belonged to the lowest socioeconomic status as defined by their asset index. Also, out of the 159 employed women, 30 % were food insecure and 10 % food insufficient.

Result of the Cronbach’s α tests show that the MSPSS tool (Cronbach’s α 0.89), the CTS2 tool (Cronbach’s α 0.85), and the HFSSM tool (Cronbach’s α 0.83) exhibit good internal consistency and reliability when used on the study sample. The multicollinearity test among independent variables also indicates that there was no multicollinearity within the regression model (VIF < 10).

In an effort to understand the factors predicting and associated with AOD use among pregnant women, we examined the association between demographic and various socioeconomic factors, life experiences, and AOD use. The logistic regression result (Table 3) shows that while women between the ages of 25–29 years were more likely to abuse alcohol and use drugs than those between the ages of 18–24 years (OR 1.35, 95 % CI 0.53–1.38), women above the ages of 29 years old were less likely to use AODs (OR 0.84, 95 % CI 0.30–1.38). Women with education levels above grade 10 were less likely to use AODs (OR 0.88, 95 % CI 0.41–0.90). Employed women were more likely to use AODs (OR 1.74, 95 % CI 1.32–1.89). Women that belonged to lower socioeconomic status as defined by the asset index were more likely to use AODs compared to those that belonged to the highest socioeconomic index. Women that were very poor and the poorest were twice as likely to use AODs as the least poor (OR 2.33, 95 % CI 1.74–2.71; OR 1.75, 95 % CI 1.53–2.78). Women that were food insecure were more likely to use AODs (OR 1.04, 95 % CI 0.42–2.59), however, women that were food insufficient were three times more likely to use AODs (OR 3.73, 95 % CI 2.323–4.29).

**Table 3:** Multivariable associations between demographic, socioeconomic, and psychosocial factors and AODs among pregnant women

Variable	Odds ratio	95 % confidence interval
Age category: 25–29 years	1.346578	0.527583–1.436942
Above 29 years	0.848982	0.302722–1.380969
Education level	0.886519	0.412610–0.904739
Employment status	1.745675	1.323856–1.896907
Asset index: poor	1.912688	0.606284–2.034089
Very poor	2.334470	1.744967–2.715419
Poorest	1.756317	1.533675–2.780012
Food insecurity	1.049968	0.424839–2.594943
Food insufficiency	3.739292	2.237959–4.294641
Partner status	1.014001	0.061702–1.121194
Relationship type	1.520949	0.629551–1.674494
Cohabiting with partner: yes	0.485067	0.414461–0.918144
Sometimes	1.554436	0.476718–2.068549
Perceived social support	0.992220	0.957972–1.027692
Planned pregnancy	0.309606	0.118785–0.806967
Positive for IPV	1.123195	0.389642–2.237756
Difficult life events	1.642240	0.269609–1.729887
MDE	1.259656	0.429228–1.696711
Any anxiety disorder	1.350071	0.538984–2.381714
Suicidal ideation	1.179614	0.457566–3.041067
History of mental health problems	2.133759	1.811383–2.611314

Women in a relationship were slightly more likely to use AODs than those who were single (OR 1.01, 95 % CI 1.06–1.12), when the type of relationship was examined, women that were in a relationship but not married were more likely to use AODs than those who were married (OR 1.52, 95 % CI 0.52–1.67). To understand further how these predictors associate with AODs, women were asked about their relationship circumstances. Even though women who were living with their partners were less likely to use AODs (OR 0.48, 95 % CI 0.44–0.91), women who partly cohabited with their partners were more likely to use AODs (OR 1.55, 95 % CI 0.47–2.06). In line with this, as women perceived more social support, based on increases in their MSPSS index, they were less likely to use AODs (0.99, 95 % CI 0.95–1.02).

Women who had planned their pregnancy were less likely to use AODs than those that had not planned their pregnancy (OR 0.30, 95 % CI 0.11–0.80). Life experiences were explored as a predictor of AOD among sampled pregnant women. Women who experienced intimate partner violence (IPV) based on the CTS2 score were more likely to use AODs (OR 1.12, 95 % CI 0.38–2.23). Also, women who experienced difficult life events (loss of employment, injuries, loss of relative, financial crisis, theft, loss of a steady relationship, etc.) were more likely to use AODs than those that have not experienced difficult life events (OR 1.64, 95 % CI 0.26–1.72).

Finally, results indicate that women who were experiencing a MDE were more likely to use AOD (OR 1.25, 95 % CI 0.42–1.69). Also women that had a current anxiety diagnosis were more likely to have used AODs than those without an anxiety diagnosis (OR 1.35, 95 % CI 0.53–1.38). Anxiety diagnoses included panic disorder, agoraphobia, social phobia or social anxiety disorder, any specific phobia, obsessive compulsive disorder, post-traumatic stress disorder, and generalised anxiety disorder. Women who indicated suicidal ideation were also more likely to use AODs (1.17, CI 0.45–3.04). Those who experienced past mental health problems were two times more likely to use AODs than those who had not experienced mental health problems in the past (OR 12.13, 95 % CI 1.81–2.61).

## Discussion

This study has confirmed the vulnerability of pregnant women in low-income, peri-urban settings to alcohol abuse and other drugs use. AOD use is closely associated with the factors that are prevalent in their living environment. The risk profile of these women indicates that there is prevailing poverty, food insecurity, intimate partner violence, and often a history of previous mental health problems. These risk factors are associated with the presence of comorbidities of AOD use with depression, anxiety and suicidality. McLanahan [56] and Gorski [4] argue that AOD use among vulnerable families results in the “production of poverty” which in turn pushes these families deeper into AOD use and poverty. This speaks to the findings of this study and suggests that pregnant women’s use of AODs places them at risk of mental health problems, poverty, and relationship difficulties, while their adverse environment places them at risk for AOD use.

Women in the mid to late twenties age group have been shown to have a higher chance of AOD use than younger or older age groups, regardless of their pregnancy status [57]. This age group has been identified as associated with higher risk-taking behaviour in South Africa [57]. Alcohol and drug use has also been shown to be associated with unprotected sex which in turn leads to pregnancy [58], and, at recognition of pregnancy, alcohol and drug use in poor women may provide a means to avoid confronting the difficulties associated with becoming pregnant [59]. Further studies in high income countries identified that women in their early to late twenties have a higher likelihood of AOD use prior to pregnancy recognition [60–62] and to alcohol abuse after confirmation of conception [59, 61]. Education, which is associated with enhanced rational knowledge and risk adverse behaviour [63, 64], was found to be negatively associated with AOD use. This indicates that the more educated a woman is, the less likely she would use AODs during pregnancy [59, 64, 65]. This study concurs with this literature.

Although over half of the women in our study were employed, more than 40 % of the sample’s earnings were below the stipulated cross-sectoral minimum wage determination as defined by the South African Department of Labour [66]. Further, having employment did not preclude these women from poverty, as over 40 % of these employed women belonged to a low socioeconomic index. The findings that women who were currently employed had more chances of using AODs contradicts findings from other studies in low-, middle-, and high-income countries [26, 67, 68]. A possible explanation for this is that those women who were employed had low incomes and low socioeconomic status. Hence, being employed did not prevent them from experiencing poverty but may have enabled sufficient financial access to procure alcohol and substances. Their pregnancy, and lack of maternity benefits, may have been added stressful life factors, predisposing them to use AODs. Food security has been identified in literature as a measure of access to enough food to meet daily dietary and energy requirements, which in turn is a measure of poverty [69, 70]. Also, results from the analysis indicate that these women had low food security as 30 % of those that were employed were food insecure and over half of those that were not employed had food insecurity. Although women that were food insecure were more likely to use AODs in this study, food insufficiency, which is a severe form of food insecurity, was found to be a stronger predictor of AOD use. This attests to the aforementioned results that illustrates that poverty and poverty-related problems predispose women to AODs, and that AOD use may contribute to their poverty.

A majority of the women in the study had a partner and were living together with their partners. Over half of the reported cases of IPV were among women cohabiting with their partners. A systematic review by Shamu et al. [71] noted that the prevalence of IPV during pregnancy ranged from 5 to 57 % in 19 studies conducted in five African countries. Although women that were cohabiting with their partners were less likely to use AODs than those living alone, those that experienced IPV were more likely to use AODs. While women that had casual partners had greater chances of using AODs, almost three quarters of those that had a casual partner lived alone. Martin et al. [72] in the USA found that the links between women’s experiences of intimate partner violence and their use of substances became stronger after pregnancy recognition, with women who experienced partner violence being more likely to use both alcohol and illicit drugs. Another explanation may be that AODs among partners may act to trigger IPV which may, in turn, create a greater likelihood of AOD use, thereby creating a vicious cycle of IPV and AODs. Makayoto et al. [73], Leonard [74], Kagle [75] and Smith [76] contend that women who use AODs are at more risk for domestic violence since their AOD use may be viewed by their partners as being inappropriate and hence may lead the partner to physically ‘‘discipline’’ the woman for her ‘‘unfeminine’’ conduct. In the Hanover Park context, there are high rates of AOD use among men, in particular the use of methamphetamine which reduces inhibitions and stimulates aggression [77, 78].

The interrelationships between perceived partner support, difficult life events, and AODs among pregnant women has been documented in literature among low income groups in predominantly high income countries [11, 79, 80]. In these settings, pregnant women who perceived lack of support from their partners and experienced adverse life events were more likely to have past mental health disorders and use AODs. Muckle et al. further demonstrated in low-income women, that the coexistence of adverse past events and past mental health problems possibly create an enabling environment for AOD use during and after pregnancy [11]. Harrison and Sidebottom found that, perceived lack of social support, and difficult live events were predictors of continued alcohol use after pregnancy recognition among women in the USA [7]. These findings resonate with the results of this study and further illustrates that the use of AODs are linked to the relationship between perceived lack of support and difficult circumstances.

Coexistence of AOD use with other forms of mental health problems like depression, anxiety, and suicidality have been identified in literature. A cohort study of women in Australia [81] found that the association of high alcohol consumption with symptoms of depression and anxiety may vary in course of a woman’s life but these associations can be confounded by low income and smoking especially during and after pregnancy. Also, a review of studies that assessed anxiety during pregnancy and postpartum among women in 27 high- and low-income countries indicate that AOD use may be used to suppress distress around pregnancy [80]. Systematic reviews of 47 studies that investigated the association between AOD use and suicidality [82–84] found that AOD use was associated with suicide for all categories of abuse and use disorders among men and women. These shows that suicide during pregnancy is disproportionately high in low income settings in both developed and developing contexts [21].

Findings from this study should be viewed in light of several design limitations. The sample size was small, limiting the ability to generalize these findings across the entire study population. The study did not assess biomedical markers for AODs by collecting urine or blood samples, thereby leaving room for underreporting of alcohol and drug use, and may have created a response bias. All data collected was self-reported since the study did not verify information like asset ownership. Several key questions were not asked in the survey. For instance, participants were not asked to report their alcohol and drug use prior to pregnancy or on the condition of assets owned. Also, HIV status was not assessed at data collection but was collected retrospectively, hence it could not be included as a predictor of AOD use. Our sampling strategy did not include non-users of antenatal services who might be particularly vulnerable to AOD use. Despite these limitations, this study has the strength of being one of the few studies that have examined the predictors of alcohol abuse and drug use among low income pregnant women in Africa. Furthermore, this study also examines inter-related, multiple risk factors that place pregnant women at greater risk of AOD use.

## Conclusions

In conclusion, our study shows that demographic, economic, and psychosocial factors play a major role in predisposing pregnant women in adverse environments, like Hanover Park, to alcohol abuse and other drug use. Further, the association between diagnosed depression and anxiety, suicidality, and AOD use among these women may largely reflect how complex environmental factors support the coexistence of multiple mental health problems. These problems are of critical concern since these women are pregnant and the outcomes for their own health and that of their infants are placed at high risk. The results have implications for planning appropriate interventions and raise questions for further research. Integrating evidence-based mental health, alcohol and drug use interventions into routine primary care settings may substantially impact outcomes for low income pregnant women. In addition, a concerted intersectoral approach is required to address the social and economic determinants of AODs and common mental disorders.

## Footnotes

1 Exchange rate of US$1.00 = 10 rands (2014) http://www.xe.com/currencytables/?from=ZAR&date=2014-06-30.

2 The first component factor is defined statistically as a weighted sum of the various assets used to assess household wealth, in order for that component to explain as much as possible of the variance observed in asset ownership between households.
